# Spatial and Temporal Habitat Use of an Asian Elephant in Sumatra

**DOI:** 10.3390/ani3030670

**Published:** 2013-07-31

**Authors:** Arnold F. Sitompul, Curtice R. Griffin, Nathaniel D. Rayl, Todd K. Fuller

**Affiliations:** 1 Conservation Science Initiative, Bogor, 16963, Indonesia; 2 Department of Environmental Conservation, 160 Holdsworth Way, University of Massachusetts, Amherst, MA 01003, USA; E-Mails: cgriffin@eco.umass.edu (C.R.G.); nathanielrayl@gmail.com (N.D.R.); tkfuller@eco.umass.edu (T.K.F.)

**Keywords:** conservation, edge, elephant, *Elephas*, forest, habitat use, Sumatra

## Abstract

**Simple Summary:**

A wild Sumatran elephant radio-monitored near a conservation center from August 2007–May 2008 used medium- and open-canopy land cover more than expected, but closed canopy forests were used more during the day than at night. When in closed canopy forests, elephants spent more time near the forest edge. Effective elephant conservation strategies in Sumatra need to focus on forest restoration of cleared areas and providing a forest matrix that includes various canopy types.

**Abstract:**

Increasingly, habitat fragmentation caused by agricultural and human development has forced Sumatran elephants into relatively small areas, but there is little information on how elephants use these areas and thus, how habitats can be managed to sustain elephants in the future. Using a Global Positioning System (GPS) collar and a land cover map developed from TM imagery, we identified the habitats used by a wild adult female elephant (*Elephas maximus sumatranus*) in the Seblat Elephant Conservation Center, Bengkulu Province, Sumatra during 2007–2008. The marked elephant (and presumably her 40–60 herd mates) used a home range that contained more than expected medium canopy and open canopy land cover. Further, within the home range, closed canopy forests were used more during the day than at night. When elephants were in closed canopy forests they were most often near the forest edge *vs*. in the forest interior. Effective elephant conservation strategies in Sumatra need to focus on forest restoration of cleared areas and providing a forest matrix that includes various canopy types.

## 1. Introduction

Since the 1990s, the wild population of Sumatran elephants (*Elephas maximus sumatranus*) has declined by approximately 35%, to an estimated 2,400–2,800 individuals [1]. Elephants occur in 25 fragmented populations in lowland areas, and all populations are considered vulnerable to continuing habitat loss from large-scale habitat conversion resulting from agriculture, human settlement, illegal logging, and forest fires [1,2,3,4]. Additionally, continually expanding anthropogenic development brings elephants into conflict with humans [5,6], often resulting in the capture and removal of elephants by the government or poisoning by local people [3]. 

Conservation strategies for Sumatran elephants focus on securing elephant habitat and mitigating human-elephant conflict [5,6,7,8]. Developing effective land conservation strategies for elephants is difficult. However, because although elephant distribution has been positively related to core forest areas [9], and to topography (*i.e.*, valleys) and forest edges [10], there is little information on the forest types used by Sumatran elephants. 

This study reports on elephant habitat use, albeit by a single satellite-tagged female, in a lowland rainforest of Sumatra. Because the population of 40–60 wild elephants believed to occur on the Seblat Elephant Conservation Center (SECC) probably range mostly as a single herd [11], we presumed for the purposes of this paper that our monitored animal’s habitat use represented that of most of the wild elephants in the study area, as well. We could be wrong, but by detailing the locations used by this elephant, we sought to identify how it used forested areas with a history of selective logging, and thus, suggest how forested areas might best be managed to sustain elephants in the future.

## 2. Methods

### 2.1. Study Area

**Figure 1:**
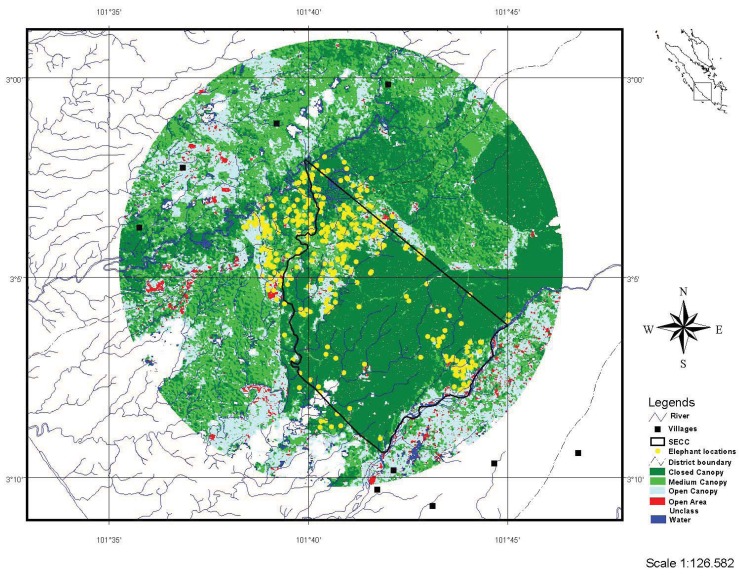
Location of study area in Bengkulu Province, Sumatra (lat 02°59'–03°11'S, long 101°34'–101°46'E), and land use within a 314-km^2^ area, including the Seblat Elephant Conservation Center.

The study was conducted in the Bengkulu Province on the west coast of Sumatra and included the SECC and surrounding forested and developed areas (314 km^2^; Figure 1). Annual rainfall typically exceeds 3,000 mm and elevations are 75–125 m above sea level with no valleys. The SECC occurs in a lowland rainforest area (Pesisir-Indrapura-Talamau ecofloristic sector; [12]) that has both original primary forest and forests that are regenerating following selective logging operations in the late 1980s. Palm oil plantations, small-scale agricultural areas, and human settlements comprise the majority of non-forested lands. In addition to 23 elephants captured as part of the government’s human-elephant conflict mitigation program and housed at the SECC, a population of 40–60 wild elephants is believed to occur at the SECC, probably occurring mostly as a single herd [11]. During the course of this study our tagged elephant was observed travelling in the elephant group by a forest ranger on elephant back during a routine patrol, and also was seen with the elephant group while they raided the adjacent palm oil plantation. With extensive agriculture and human settlements surrounding much of the SECC, there is much human-elephant conflict in the area; during 2008–2009 in adjacent palm oil plantations, at least 17 conflict incidents occurred and ~2,800 young palm trees were damaged [13]. 

### 2.2. Telemetry

On August 25, 2007, one adult (~25 years old) wild female elephant was darted from the back of an elephant herd [14], fitted with a GPS collar (Satellite Collar-DMR800, Africa Wildlife Tracking, Inc, Pretoria, South Africa), and observed until fully recovered. The duty cycle of the unit was set to download three GPS fixes per 24-hour period (0100, 0900, 1700 h) and a 9-minute GPS login time period was used for each monitoring interval. Through May 14, 2008 we were successful in obtaining about half of the attempted fixes, and location error from a test collar was 2.3m (n = 26). We plotted all elephant locations on LANDSAT Thematic Mapper (TM) satellite images, determined location vegetation types, and then entered data into a GIS database (ArcView GIS version 3.3; ESRI). Ad hoc ground checks during surveys matched our classifications. 

2.3 Land Cover Classification

We created a land cover map for the study area using Landsat TM 2005 satellite images. We assigned land cover classes to the image using supervised classification techniques with ERDAS IMAGINE 8.4. Initially, we identified 20 categories based on reflectance, excluding bands 6 and 8. We re-classified these 20 categories into five land cover types: closed canopy forest, medium canopy, open canopy, open area and water (Table 1). These five broad categories were selected considering the accuracy of land cover classification, ecological significance for elephants and subsequent habitat management by resource managers. To avoid problems of including habitat that the GPS-tagged elephant may not have had access to in the study area [15], we restricted the analyses to all available habitats within a 10-km radius (314 km^2^) of a central circular point statistic of all elephant location data (Figure 1). This area included the elephant’s ~97 km^2^ home range [16], as well as areas 2–7 km immediately adjacent. 

**Table 1:** Habitat descriptions and abundance in the elephant study area at Seblat, Bengkulu, Sumatra.

Habitat category	Description	Proportion of study area (km^2^)
Closed canopy forest	Area with closed canopy forest and dense tree vegetation. Includes some mature (>10 years) palm plantation.	0.455 (143)
Medium canopy	Area with broken canopy or rare standing tree vegetation. This area also mainly covered with secondary vegetation or tall shrub vegetation including bamboo vegetation. Includes some young palm plantation.	0.283 (89)
Open canopy	Area with no tree vegetation and dominated with secondary vegetation, shrub or Alang-alang *(Imperata cylindrica)*. Includes agricultural land.	0.226 (71)
Open area	This area mostly bare ground or area with rare small vegetation, mostly grasses (e.g., Poaceae family) or small shrubs. Includes human settlement.	0.017 (5)
Water	Water body including ponds, stream or river.	0.019 (6)

### 2.4. Habitat Use Analysis

To evaluate elephant habitat use, we first examined second order habitat selection (selection of the entire home range within the larger defined study area) by delineating an estimate of the elephant's home range (358 locations) using a 95% fixed kernel density estimator [17] with the reference bandwidth. We used the Hawth’s Tools extension [18] for ArcGIS 9.3 (ESRI, Redlands, CA, USA) to generate kernels. We then followed procedures of Manly *et al.* [19] and calculated a Resource Selection Ratio (ŵi) by comparing the observed number of elephant GPS locations in each habitat type to habitat availability (expected use based upon proportions of each habitat in study area). The resource selection ratios (ŵi) were then standardized and chi-square goodness of fit tests used to identify if there was significant use of a habitat category. Chi-squared values were then compared with a chi-square distribution statistic with k-1 degrees of freedom. When a significant difference was detected, we used Bonferroni Z statistic to determine habitat selection ratios (the habitat type used more or less frequently than expected (a = 0.05)). If the confidence interval for ŵi resource selection ratios did not contain a value of 1, then selection for that habitat was inferred.

For third order habitat selection (a comparison of habitats used within the home range with those available in the home range) we used Compositional Analysis [20] to evaluate differences in nocturnal *vs*. diurnal habitat use (locations at 0100 h for nocturnal activity *vs.* 0900 h for diurnal). For these analyses, used habitats were estimated only from locations (nocturnal and diurnal, respectively) obtained during the first five days of each month (n = 60) to ensure independence among months [20]. We assessed significant deviation from random use (habitat composition in monthly kernel ranges) and ranked habitat types from most to least used at each level of habitat selection [21] using multivariate analysis of log ratio test [20]. Prior to the compositional analysis, we replaced zero values with the value of 0.001%, an order of magnitude less than the smallest recorded nonzero proportion [22].

### 2.5. Distance to Edge

To evaluate the relationship between elephant locations and forest edge we compared the distances of both elephant and random locations within the elephant's home range to the forest edge, depending on whether the elephant was inside or outside of the forest. We defined the forest edge as the border between the “closed canopy forest” cover class and “other” land cover classes; these included the pooled “medium canopy land cover”, “open canopy land cover”, “open area”, and “water” land cover classes. We estimated the Euclidean distance to the forest edge for elephant locations and for 1,000 randomly generated points within the 95% fixed kernel home range. Distances were binned into five classes (0–49 m, 50–99 m, 100–149 m, 150–199 m, and >200 m) for locations within the “closed canopy forest” cover class and locations within the “other” cover class. We used Pearson’s chi-square test to analyze the difference in distance to edge between the binned elephant locations and the binned random locations in each cover class (closed canopy forest, other). 

## 3. Results

Manly’s resource selection analyses indicated that the home range of the single female elephant had habitat composition that differed significantly from availability (*χ*^2^ = 21.512, df = 4, *P* < 0.001). Medium canopy and open canopy were more common in the home range, and closed canopy forest, open area and water habitat categories occurred less than expected, but not significantly so (Table 2). The third order compositional analysis of the female’s locations within the home range differed from random use for diurnal (Λ = 0.0028, χ^2^ = 47.052, randomized *P* < 0.001) and nocturnal (Λ = 0.07, χ^2^ = 21.242, randomized *P* < 0.001) locations; this female used open canopy land cover more during the night *vs.* medium canopy land cover and closed canopy forest more during the day (Table 3).

**Figure 2:**
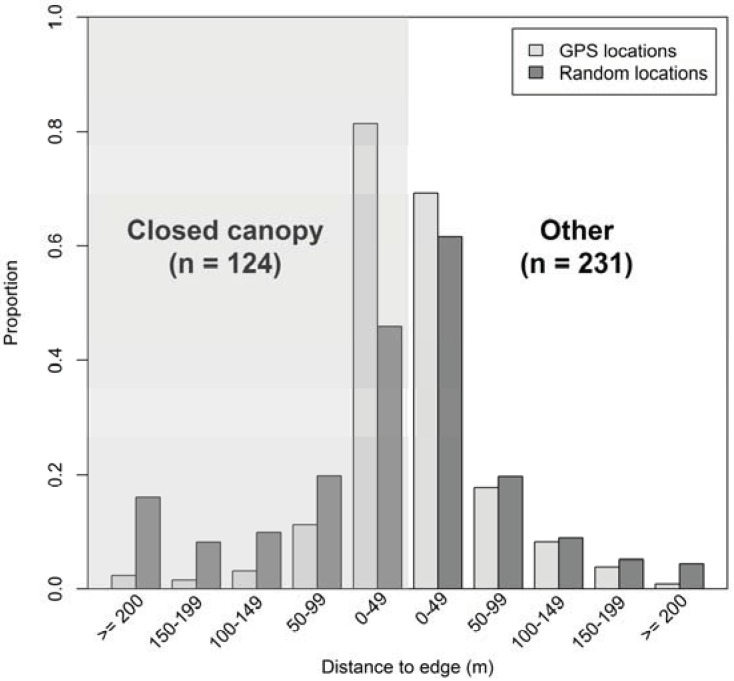
Proportions of elephant locations (total n = 355) and random locations (n = 1,000) in categories of the Euclidean distance (m) to the forest edge, depending on whether elephants were within or outside (“other”) closed canopy forest (shaded portion).

Of 355 elephant locations within the 95% fixed kernel home range, 124 locations were within the closed canopy forest. When in the forest, elephants were located much more often near the edge and less often in the interior (Figure 2; Pearson’s chi-square: χ^2^ = 37.254, df = 4, *P* < 0.001). The median distance of elephant locations in the forest to the forest edge was 22 m (range = <1–452 m), and >80% of all locations within the forest were <50 m from the edge. In contrast, the median distance of random locations to the forest edge was 56 m (range = <1–695 m), and <50% of all random locations within the closed canopy forest were <50 m from the forest edge. 

We detected no differences in the distance to the forest edge between 231 elephant locations and random locations outside the closed canopy forest (Pearson’s chi-square: χ^2^ = 7.222, df = 4, *P* = 0.125). The median distance of elephant locations outside the closed canopy forest to the forest edge was 28 m (range = <1–270 m); the median distance of random locations outside the closed canopy forest to the forest edge was 33m (range = <1–348 m).

**Table 2:** Resource selection indices with Bonferroni confidence intervals for habitats used by an adult female elephant radio-monitored from August 25, 2007–May 14, 2008, near the Seblat Elephant Conservation Center, Bengkulu Province, Sumatra.

Habitat	Population proportion (π)	Sample count (u)	Used sample proportion (o)	Expected count(π *u_t_)	Selection ratio (ŵ)	Manly standardize Index (B)	Bonferroni confidence limits		Selection level	Sig P < 0.05
							Lower	Upper		
Closed canopy forest	0.4549	126	0.353	162.413	0.7758	0.1687	0.75	2.46	"-"	NS
Medium canopy	0.2827	135	0.378	100.929	1.3376	0.2908	1.32	4.30	"+"	S
Open canopy	0.2264	87	0.244	80.837	1.0762	0.2340	1.06	3.46	"+"	S
Open area	0.0174	5	0.014	6.220	0.8038	0.1748	0.80	2.60	"-"	NS
Water	0.0185	4	0.011	6.601	0.6059	0.1317	0.61	1.96	"-"	NS
	1	357			4.5994	1				

**Table 3:** Nocturnal (**A**) and diurnal (**B**) habitat ranking matrix using 3rd order compositional analyses for five habitats (bold) used by an adult, female elephant from August 25, 2007–May 14, 2008, at Seblat, Bengkulu, Sumatra.

(A)	Closed canopy forest	Open area	Medium canopy	Open canopy	Water	Rank
Closed canopy forest		+++	---	---	+++	3
Open area	---		---	---	-	5
Medium canopy	+++	+++		-	+++	2
Open canopy	+++	+++	+		+++	1
Water	---	+	---	---		4
(B)	Closed canopy forest	Open area	Medium canopy	Open canopy	Water	Rank
Closed Canopy forest		+++	---	+	+++	2
Open area	---		---	---	-	5
Medium canopy	+++	+++		+	+++	1
Open canopy	-	+++	-		+++	3
Water	---	+	---	---		4

* Higher ranking (1 is highest) indicates greater use compared to availability. Within the matrix, “+” represents the row habitat is preferred over the column habitat, whereas “-“ represents the opposite. Triple signs represent significant deviation from random at *P* < 0.05.

## 4. Discussion

Overall, the female elephant in the SECC used medium canopy and open canopy land cover more often than expected. These results were consistent with results of analyses of elephant locations and a remotely sensed enhanced vegetation index (EVI; [16]). High use of medium canopy and open canopy land cover may be related to food availability. Medium canopy land cover appeared to have abundant browse including bamboos and rattan, while open canopy land cover had abundant grasses (e.g., Poaceae family). Chen *et al.* [23] reported reduced availability of many important elephant food plants such as *Dendorcalamus* spp. (Poaceae), *Musa acuminata* (Musaceae) and *Microstegium ciliatum* (Poaceae) with the loss of secondary and early successional forests in Xishuangbanna Nature Reserve in China. The lower rank of closed canopy forests in elephant use in Seblat may be related to the relatively low abundance of elephant foods in closed canopy forests as indicated by the reportedly low densities of elephants in tropical forests [3,23,24]. The low use of open area and water habitats probably reflects their infrequent use by elephants for water and minerals.

The high use of closed canopy habitat during the day in contrast to night is probably related to thermal regulation and the shade provided by the closed canopy during the day. Thermoregulation has also been observed in other herbivore species where shaded areas are preferred when solar radiation is high [25,26]. Valeix *et al.* [27] reported that several large ungulates occurred more often in closed canopy forest during the hottest period of the day, shortening the time period of access to water because they avoided staying in open areas to protect them from direct solar radiation.

When located outside of closed canopy forests, our elephant was not particularly attracted to the forest edge, but when located in the forest, it was less often in the forest interior and more often near the edge. This is in contrast to findings from camera trap studies in southern Sumatra [9] where elephants seemingly avoided forest boundaries (up to 3 km), but similar to perceived elephant habitat distribution in northern Sumatra [10]. In our study area, which is highly fragmented and seemingly well-protected, the influence of forest edge was likely not related to specific ecological edge effects on vegetation (e.g., [28,29]), but rather to the probability that forage in non-forest areas was abundant, human "predation" in non-forest areas was not important [29,30], and when elephants used forested areas for resting, they did so very near the forest edge. 

## 5. Conclusions

Sumatran elephants likely use a variety of forest types, ranging from open land cover to closed canopy forests. Open and medium canopy land covers are probably the most important habitats for feeding, whereas closed canopy forests may be most important for thermoregulation. However, elephant distribution is also influenced by human activities in some areas; when forests are completely replaced by agriculture, elephant conflicts are likely to arise and elephants may be less likely to use even closed-canopy forest near (at the edge of or border with) such human activities [9], much less more open canopy land cover, even though forage may be better there. 

Because our elephant spent much of its time in areas of regenerating forest areas, and not in closed canopy forest interiors, the opportunities to conserve previously logged areas as elephant habitat seem important. Also, the elephant tended to avoid open areas; thus, effective elephant conservation strategies in Sumatra likely need to focus on forest restoration of cleared areas and providing a forest matrix that includes various canopy types. Protecting only closed forested habitat likely is neither sufficient nor prudent in order to effectively conserve elephants in Sumatra. Degraded forests might, under some circumstances, also need to be considered as part of viable elephant habitat, particularly where forest cover is reduced, access by humans is limited and conflicts with agriculture can be mitigated. Further research is needed to compare the results of this study to that from other elephant populations in Sumatra where habitat conditions differ (e.g., ex-logging concession areas). This might indicate ex-logging concessions should be maintained instead of converting them in to large-scale palm oil plantation or mining. Finally, effective elephant conservation strategies likely need to consider the movements and behaviors of elephants where habitat mixes and configurations differ, something we were not able to address with our limited sample.
